# The Salts of Quinia

**Published:** 1843-04

**Authors:** 


*The Salts of Quinia.*—Prince Lucien Bonaparte has been making further researches on these medicinal agents. We have already once alluded to his experiments. He now recommends the employment in practice of both lactate and valerianate of quinia in preference to the sulphate, the latter not producing those functional derangements in the nervous system which the sulphate sometimes causes; and the former on account both of its greater solubility and more energetic action. The fact, established by various physicians in the Roman Maremme, that quinia alone, or its hydrate, is more efficacious as a remedy for intermittents than the sulphate, the prince considers due to its being converted into a lactate by the lactic acid of the gastric juice. This opportunity may be taken to mention (see Gaz. des Hopitaux) that attempts have been made to combine quinia with ferrocyanic acid, and a substance entitled *hydro-ferrocyanate of quinine* has crept into pretty extensive use among French practitioners. But Pelouze has ascertained that this substance is in reality nothing more than quinine mechanically mixed with a little Prussian blue, the consequence of spontaneous decomposition of the acid.—*London Lancet*, Jan. 28, 1843.

